# Succinyl-CoA-based energy metabolism dysfunction in chronic heart failure

**DOI:** 10.1073/pnas.2203628119

**Published:** 2022-10-06

**Authors:** Shingo Takada, Satoshi Maekawa, Takaaki Furihata, Naoya Kakutani, Daiki Setoyama, Koji Ueda, Hideo Nambu, Hikaru Hagiwara, Haruka Handa, Yoshizuki Fumoto, Soichiro Hata, Tomoka Masunaga, Arata Fukushima, Takashi Yokota, Dongchon Kang, Shintaro Kinugawa, Hisataka Sabe

**Affiliations:** ^a^Department of Cardiovascular Medicine, Hokkaido University Graduate School of Medicine, Sapporo, 060-8638 Japan;; ^b^Department of Molecular Biology, Hokkaido University Graduate School of Medicine, Sapporo, 060-8638 Japan;; ^c^Department of Lifelong Sport, School of Sports Education, Hokusho University, Ebetsu, 069-8511 Japan;; ^d^Department of Clinical Chemistry and Laboratory Medicine, Kyushu University, Fukuoka, 812-8582 Japan;; ^e^Cancer Precision Medicine Center, Japanese Foundation for Cancer Research, Tokyo, 135-8550 Japan;; ^f^Department of Cardiovascular Medicine, Faculty of Medical Sciences, Kyushu University, Fukuoka, 812-8582 Japan;; ^g^Clinical Laboratories, Kyushu University Hospital, Fukuoka, 812-8582 Japan;; ^h^Division of Cardiovascular Medicine, Research Institute of Angiocardiology, Faculty of Medical Sciences, Kyushu University, Fukuoka, 812-8582 Japan;; ^i^Institute for Genetic Medicine, Hokkaido University, Sapporo, 060-8638 Japan

**Keywords:** heart failure, succinyl-CoA, mitochondria, oxidative phosphorylation, 5-aminolevulinic acid

## Abstract

Heart failure (HF) is a leading cause of death and repeated hospitalizations and often involves cardiac mitochondrial dysfunction. However, the underlying mechanisms largely remain elusive. Here, using a mouse model in which myocardial infarction (MI) was induced by coronary artery ligation, we show the metabolic basis of mitochondrial dysfunction in chronic HF. Four weeks after ligation, MI mice showed a significant decrease in myocardial succinyl-CoA levels, and this decrease impaired the mitochondrial oxidative phosphorylation (OXPHOS) capacity. Heme synthesis and ketolysis, and protein levels of several enzymes consuming succinyl-CoA in these events, were increased in MI mice, while enzymes synthesizing succinyl-CoA from α-ketoglutarate and glutamate were also increased. Furthermore, the ADP-specific subunit of succinyl-CoA synthase was reduced, while its GDP-specific subunit was almost unchanged. Administration of 5-aminolevulinic acid, an intermediate in the pathway from succinyl-CoA to heme synthesis, appreciably restored succinyl-CoA levels and OXPHOS capacity and prevented HF progression in MI mice. Previous reports also suggested the presence of succinyl-CoA metabolism abnormalities in cardiac muscles of HF patients. Our results identified that changes in succinyl-CoA usage in different metabolisms of the mitochondrial energy production system is characteristic to chronic HF, and although similar alterations are known to occur in healthy conditions, such as during strenuous exercise, they may often occur irreversibly in chronic HF leading to a decrease in succinyl-CoA. Consequently, nutritional interventions compensating the succinyl-CoA consumption are expected to be promising strategies to treat HF.

## Significance

Metabolic changes frequently occur in patients with chronic heart failure (HF). Therefore, detailed identification of these metabolic changes, and complementing them, will provide new therapeutic approaches. Here, using a mouse model, we demonstrated that succinyl-CoA levels are reduced in the myocardial mitochondria of hearts undergoing chronic HF, and this reduction impairs mitochondrial oxidative phosphorylation capacity. We identified increased heme synthesis as a cause of this succinyl-CoA reduction and demonstrated a method that can compensate substantially for the increased succinyl-CoA consumption. Reduction in succinyl-CoA levels has also been reported in HF patients. Our results provide an academic basis for the development of new treatment methodologies against HF, which target the altered metabolic activities that occur in HF by nutritional interventions.

Mitochondrial dysfunction is closely associated with the development of heart failure (HF), and metabolic dysfunction, including that of the tricarboxylic acid (TCA) cycle, which is a core metabolic pathway for producing ATP, is known as the main cause (1–3). Recent studies have shown that the selective accumulation of succinate, an intermediate of the TCA cycle, during acute ischemia in the mouse heart is a major cause of reperfusion injury (4–6). On the other hand, how the TCA cycle and its associated metabolic pathways respond to chronic HF remains largely unclear. Here, we addressed this question with the aim of understanding the metabolic basis of the mitochondrial dysfunction occurring in chronic HF. We used a mouse model of HF, in which MI was induced by permanent left anterior descending coronary artery ligation (7, 8). Mice with permanent coronary artery ligation (hereafter referred to as MI mice) generally start to show HF symptoms 7 d after the ligation (7, 8), which we confirmed again in this study (*SI Appendix*, Fig. S1). Sham-operated mice were used as a control (7, 8). We used these mice 28 d after surgery in all the following analyses, unless otherwise described. The myocardium is generally subdivided into three areas in MI mice: infarcted area, border zone, and noninfarcted area; and metabolic responses may be different depending on these subdivided areas. In this study, we used noninfarcted, surviving areas in our biochemical analyses and excluded the infarcted areas and the border zones between the infarcted and noninfarcted areas.

## Results

### Succinyl-CoA Is Decreased in Chronic HF.

**Fig. 1.**
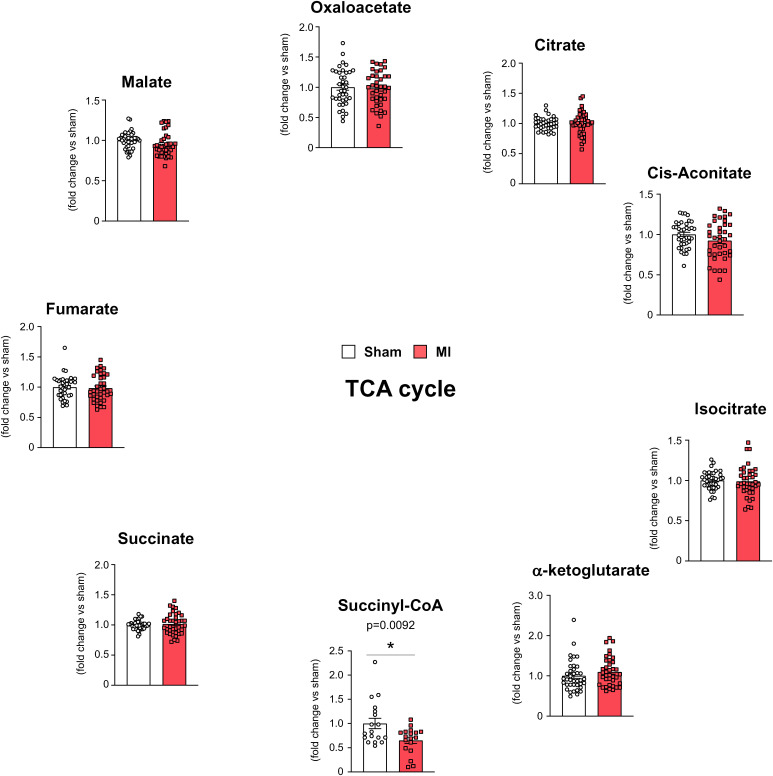
Selective reduction of succinyl-CoA among the TCA cycle metabolites in cardiac muscle during chronic HF. Relative levels of the TCA cycle metabolites in cardiac muscles isolated from myocardial infarction (MI) mice, compared with those from sham mice, 28 d after surgery. Each data point in the dot plot represents one individual mouse sample (*n* = 38 for both groups for all metabolites, except for succinyl-CoA in which *n* = 18 for both groups). Concentrations of succinyl-CoA in these samples were 2.2–50 pmol/mg wet weight. Data are shown as the mean ± SEM. Significances between groups were tested using the unpaired *t* test, and indicated by asterisks (*P < 0.05).

To investigate the possible metabolic changes that occur in the mouse heart during chronic HF, we first analyzed the levels of TCA cycle intermediates. Liquid chromatography-mass spectrometry (LC-MS) analysis demonstrated a statistically significant decrease in succinyl-CoA levels in the cardiac muscles of MI mice compared with those of sham mice, although there were some individual variations in both MI mice and sham mice (Fig. 1). The majority of succinyl-CoA is produced by the TCA cycle (9). Levels of other TCA cycle intermediates, including succinate, did not show a statistically significant difference between MI mice and sham mice (Fig. 1). Therefore, metabolic changes that frequently occur in the noninfarcted, surviving myocardium of the mouse heart under chronic HF may be substantially different from those that occur in the ischemic myocardium under acute HF (4–6).

### Succinyl-CoA Reduction Impairs OXPHOS.

**Fig. 2.**
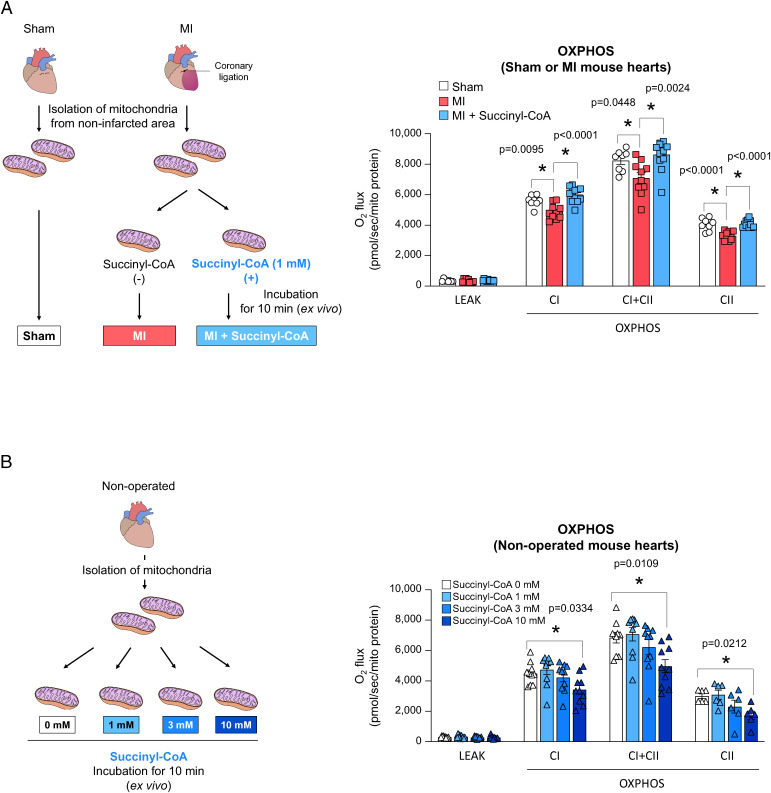
Reduced succinyl-CoA level impairs OXPHOS activities of myocardial mitochondria during chronic HF. (*A*) Experimental scheme to measure myocardial mitochondrial OXPHOS activities of MI mice and sham mice, and their responses to the addition of succinyl-CoA (*Left*); and the actual results (*Right*). (*B*) Experimental scheme to measure myocardial mitochondrial OXPHOS activities in nonoperated mice in response to the addition of succinyl-CoA (*Left*) and the actual results (*Right*). In (*A, Right*) and (*B, Right*), each data point in the dot plot represents one individual mouse sample. Data are shown as the mean ± SEM. Data were analyzed by one-way analysis of variance (ANOVA) with the Tukey post hoc analysis (*A*) or one-way ANOVA with the Dunnett post hoc analysis (*B*). Significances between groups are indicated by asterisks (*P < 0.05). LEAK, leak state; CI, mitochondrial complex I; CII, mitochondrial complex II; OXPHOS, oxidative phosphorylation.

Reduced mitochondrial OXPHOS capacity is frequently associated with the development of HF (1). We then addressed whether the decreased succinyl-CoA levels in MI mice impair mitochondrial OXPHOS capacity. Both complex I (CI)-linked (i.e., NADH-driven CI/complex III [CIII]/complex IV [CIV] supercomplex) and complex II (CII)-linked (i.e., FADH-driven CII/CIII/CIV chain) OXPHOS capacities are reported to be decreased in the myocardial mitochondria of MI mice (10), which we hereby confirmed (Fig. 2*A*). We then found that the preincubation of myocardial mitochondria isolated from MI mice with 1 mM succinyl-CoA significantly increased their CI- and CII-linked OXPHOS capacities to levels almost comparable to those of sham mice (Fig. 2*A*). Myocardial mitochondria isolated from nonoperated mice did not show such positive response to succinyl-CoA (Fig. 2*B*), and 1–10 mM succinate also did not increase OXPHOS (*SI Appendix*, Fig. S2). Therefore, these results indicate that the decrease in succinyl-CoA level in the cardiac muscle of mice during chronic HF of MI mice is likely to contribute to the reduced CI- and CII-linked OXPHOS capacities of the failing heart.

### Levels of Enzymes Associated with Succinyl-CoA Metabolism Changes in Chronic HF.

**Fig. 3.**
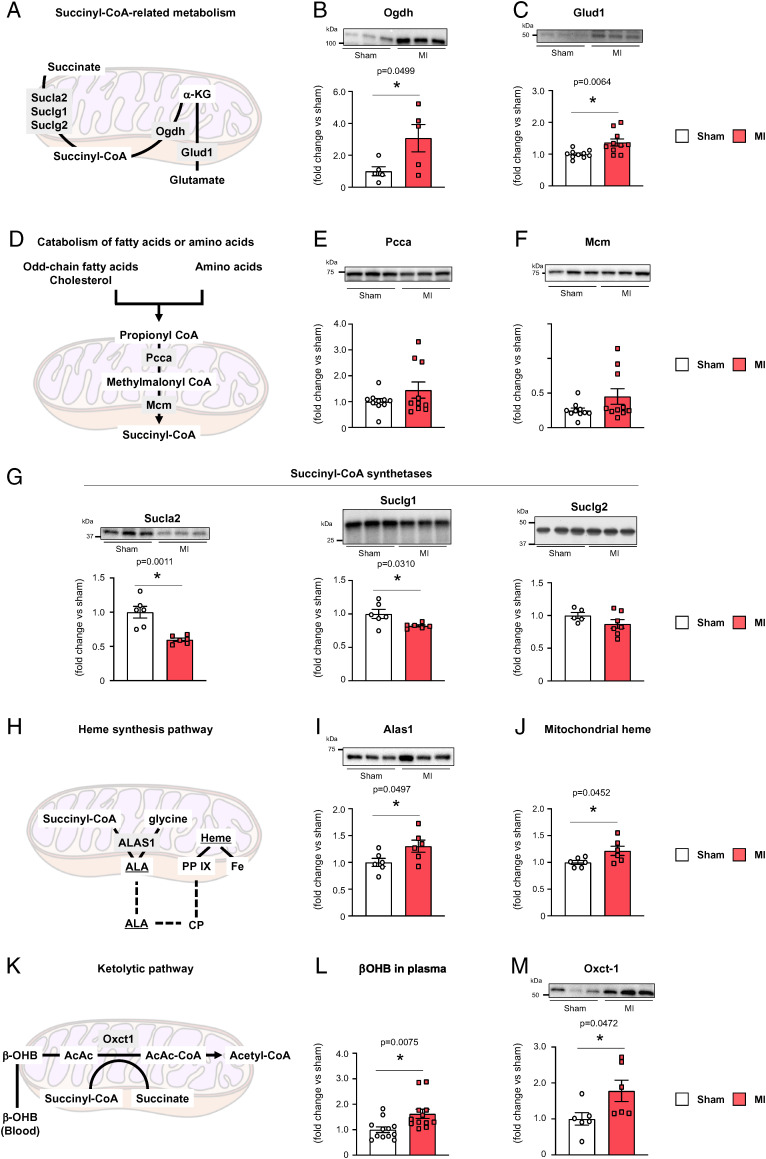
Enzyme level changes in myocardial mitochondria during chronic HF promote heme synthesis and ketolysis, and down-regulate the incorporation of succinyl-CoA into the TCA cycle. (*A–C*) Succinyl-CoA metabolism in the TCA cycle (*A*) and relative protein levels of Ogdh (*B*) (*n* = 5) and Glud1 (*C*) (*n* = 10). (*D–F*) Propionyl-CoA-based succinyl-CoA synthesis in the mitochondrion (*A*) and relative protein levels of Pcca (*E*) (*n* = 10) and Mcm (*F*) (*n* = 10). (*G*) Relative protein levels of the succinyl-CoA synthetase subunits Sucla2 (*Left*, *n* = 6), Suclg1 (*Middle*, *n* = 6), and Suclg2 (*Right*, *n* = 6). (*H–J*) Heme synthesis pathway in the mitochondrion (*H*) and relative protein levels of Alas1 (*I*) (*n* = 6) and relative amounts of heme (*J*) (*n* = 6). CP, coproporphyrinogen-III; PPIX, protoporphyrin IX. (*K–N*) Ketolytic pathway in the mitochondrion (*K*), relative amounts of β-OHB in plasma (*L*) (*n* = 12), and relative levels of Oxct1 in mitochondria (*M*) (*n* = 6). All assays were performed using isolated myocardial mitochondria, except for (*L*) (blood plasma). In (*B*), (*C*), (*E*–*G*), (*I*), and (*M*), representative results of each immunoblot blot are shown in the upper panels. Each data point in the dot plot represents one individual mouse sample. Significances between groups were tested using the unpaired *t* test, and are indicated by asterisks (*P < 0.05). Full-size CBB staining scans of the immunoblots are shown in *SI Appendix*, Fig. S6.

We then sought to understand the molecular mechanisms that may affect succinyl-CoA levels in the cardiac muscle of MI mice. Various mechanisms may be involved in the regulation of enzyme activities, and it is often difficult to clarify them precisely in experiments. For example, tracer experiments in which only certain metabolic intermediates are administered may interfere with the potential allosteric regulation of enzymes by metabolites. Thus, as a possible and simple way to investigate the metabolic changes occurring in MI mice, we analyzed whether the protein levels of enzymes associated with succinyl-CoA were altered. The E1 component of the 2-oxoglutarate dehydrogenase (Ogdh) complex generates succinyl-CoA from α-ketoglutarate (αKG) in the TCA cycle (see Fig. 3*A*). We found that there is a statistically significant increase in the protein levels of Ogdh in MI mice compared with sham mice, although, as in the case of succinyl-CoA levels, there were individual variations to some extent (Fig. 3*B*). There was also a statistically significant increase in the protein levels of glutamate dehydrogenase 1 (Glud1), which catalyzes the reversible reaction of glutamate to αKG (Fig. 3*C*). On the other hand, there was no statistical difference in the protein levels of propionyl-CoA carboxylase alpha (Pcca) and methylmalonyl-CoA mutase (Mcm), which synthesize succinyl-CoA from specific fatty acids and amino acids (Fig. 3 *D–F*). Succinyl-CoA synthetase catalyzes the reversible reaction of succinyl-CoA to succinate in the TCA cycle (see Fig. 1*A*). Levels of the noncatalytic ADP-specific β subunit (Sucla2) and the catalytic α subunit (Suclg1) of this enzyme were significantly decreased in MI mice compared with sham mice (Fig. 3*G*); the decrease in the catalytic subunit was only slight, although statistically significant. On the other hand, levels of the noncatalytic GDP-specific β subunit (Suclg2) did not show a statistically significant difference between MI mice and sham mice (Fig. 3*G*). Therefore, cardiac mitochondria may suppress the ADP/ATP-associated activity of succinyl-CoA synthetase, while maintaining GDP/GTP-associated activity almost intact in MI mice. In any case, however, changes in protein levels of the several enzymes described above may all be advantageous for increasing succinyl-CoA levels. Therefore, these changes do not appear to be the direct cause of the selective reduction in succinyl-CoA levels that we observed above in MI mice.

In addition to the forward reaction of succinyl-CoA synthetase, there are several other pathways that utilize succinyl-CoA in the mitochondrion. Heme is essential for mitochondrial OXPHOS. Mitochondrial heme synthesis is initiated from the synthesis of 5-aminolevulinic acid (5-ALA), which is synthesized from succinyl-CoA and glycine by the mitochondrial rate-limiting enzyme 5′-aminolevulinate synthase 1 (Alas1) (11, 12) (see Fig. 3*H*). We found that the protein levels of Alas1, as well as heme levels in the myocardial mitochondria of MI mice are statistically significantly higher than those in sham mice (Fig. 3 *I* and *J*). Moreover, cardiac muscle can utilize ketone bodies in a process in which succinyl-CoA is consumed by 3-oxoacid CoA-transferase 1 (Oxct1) to convert acetoacetate into acetoacetyl-CoA (i.e., ketolysis) (see Fig. 3*K*). In the liver, acetoacetate is converted to β-hydroxybutyrate (β-OHB), which is then released into the blood (13, 14); and subsequently, the cardiac muscle incorporates β-OHB to generate acetoacetate. Ketone body production in the liver and ketone body utilization in the heart are known to be increased in HF patients and animal models (13, 14). We found that the levels of β-OHB in plasma and Oxct1 in myocardial mitochondria were statistically significantly increased in MI mice compared with sham mice (Fig. 3 *L* and *M*). Therefore, collectively, heme synthesis and ketolysis, and protein levels of Alas1 and Oxct1 may be increased in myocardial mitochondria during chronic HF of MI mice.

### Excess Succinyl-CoA Consumption Is Antagonized by 5-ALA, Resulting in the Inhibition of HF Progression.

**Fig. 4.**
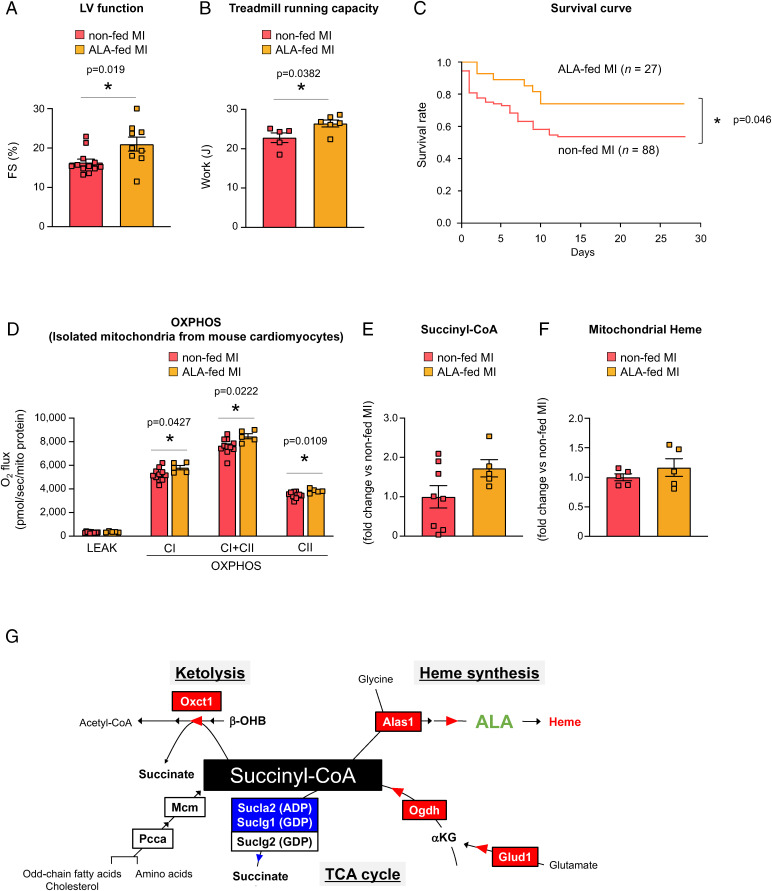
Therapeutic effects of 5-ALA against MI-induced HF. (*A–C*) Effects of 5-ALA on HF symptoms were analyzed by administering MI mice with or without 5-ALA in their drinking water for 4 wk, starting immediately after the permanent coronary artery ligation; and then measuring their LV function (i.e., the percent fractional shortening [%FS]) (*A*), treadmill running capacity (*B*), and survival (*C*). (*D–F*) Effects of 5-ALA on OXPHOS activities (*D*), relative succinyl-CoA levels (*E*), and relative heme levels (*F*), measured in myocardial mitochondria isolated from ALA-fed or nonfed MI mice. Each data point in the dot plots represents one individual mouse sample. In (*A*), (*B*), and (*D*–*F*), data are shown as the mean ± SEM. Significances between groups were tested using the unpaired *t* test (*A*, *B*, and *D*–*F*) or two-sided log-rank test (*C*), and are indicated by asterisks (*P < 0.05). (*G*) A proposed model of the metabolic shifts in myocardial mitochondria during chronic HF, whereby we demonstrated that 5-ALA administration to mice can improve succinyl-CoA levels and OXPHOS activities, as well as heart function, and prolong survival.

We then hypothesized that a reason for the decrease in succinyl-CoA levels in MI mice may be the excessive consumption of succinyl-CoA for heme synthesis and/or ketolysis. The molecule 5-ALA is an intermediate in the pathway from succinyl-CoA to heme synthesis. We tested our hypothesis by administering 5-ALA to MI mice, from immediately after coronary ligation for 4 wk, by adding 5-ALA to their drinking water. The administration of 5-ALA significantly improved the left ventricular (LV) function and treadmill running capacity (i.e., systemic exercise capacity) of MI mice measured at 4 wk after the coronary ligation and also improved their survival (Fig. 4 *A–C*). The enlargement of LV-end-diastolic diameter, a parameter of LV remodeling, also tended to be prevented in MI mice treated with 5-ALA (*SI Appendix*, Fig. S3). Molecularly, both CI- and CII-linked OXPHOS capacities of myocardial mitochondria were significantly improved in MI mice treated with 5-ALA (Fig. 4*D*). The administration of 5-ALA also appeared to sufficiently restore succinyl-CoA levels of the myocardial mitochondria of MI mice, although a statistically significant difference was not observed between MI mice with and without 5-ALA treatment (Fig. 4*E*). This may be primarily owing to a certain degree of individual variation in the succinyl-CoA levels of MI mice that were not treated with 5-ALA. On the other hand, 5-ALA treatment did not further increase mitochondrial heme levels in MI mice (Fig. 4*F*). We then tested the effects of 5-ALA in sham mice for 4 wk from immediately after the sham operation, by adding 5-ALA to their drinking water. The administration of 5-ALA did not alter cardiac parameters, or treadmill running capacity of sham mice measured at 4 wk after the sham operation (*SI Appendix*, Fig. S4). Taken together, 5-ALA might be effective, to certain extents, for the prevention of HF progression after MI. Moreover, these results support the hypothesis that the decreased succinyl-CoA level in MI mice is owed, at least in part, to the excessive consumption of succinyl-CoA for heme synthesis.

### Reduced Mitochondrial Protein Succinylation in MI Mice.

Succinyl-CoA is a source of protein succinylation (15, 16). Succinylation occurs predominantly on mitochondrial proteins in the heart, and may affect some of their enzymatic activities (17). Decreased protein succinylation in the cardiac myofibrils of failing human hearts was reported previously (18). We hence finally investigated the succinylation of myocardial mitochondria proteins in MI mice. The overall levels of protein succinylation were significantly lower in the myocardial mitochondria isolated from MI mice than those from sham mice, as reported previously (18). Incubation of the mitochondria of MI mice with succinyl-CoA significantly increased overall protein succinylation levels (*SI Appendix*, Fig. S5 *A* and *B*). Therefore, the succinylation of mitochondrial proteins in cardiac muscle appeared to also be reduced overall in chronic HF, and succinyl-CoA may be involved in this phenomenon.

We then investigated succinylation of individual mitochondrial proteins (*SI Appendix*, Fig. S5 *C*–*H*); and found that succinylation of some proteins was increased in MI mice compared with those of sham mice, as has also been reported previously (18). For example, the succinylation of some TCA cycle enzymes and membrane proteins tended to be higher in MI mice than in sham mice, and interestingly, succinylation levels of these proteins tended to be decreased upon the addition of succinyl-CoA (Fig. 3 *F* and *G*). Moreover, some other proteins, such as succinyl dehydrogenase complex iron sulfur subunit B (Sdhb), showed more complicated changes in MI mice (*SI Appendix*, Fig. S5*E*). Therefore, it is possible that some succinylation/desuccinylation processes of mitochondrial proteins are impaired in chronic HF, and, moreover, that such alterations cannot be restored easily by the addition of succinyl-CoA. On the other hand, succinylation of some proteins is known to proceed nonenzymatically in the presence of succinyl-CoA (17). Thus, it is also possible that the succinylation of some mitochondrial proteins is not precisely regulated and does not occur at a specific stoichiometry. In any case, it remains to be clarified whether the succinylation of various mitochondrial proteins has substantial effects on cardiac function.

## Discussion

In this study, using a well-established MI mouse model, we demonstrated that the selective decrease in myocardial succinyl-CoA levels is a prominent feature of chronic HF, and that this decrease is associated with a reduction in mitochondrial OXPHOS capacity. Consistently, a recent report analyzing clinical heart specimens suggested the dysfunction of succinyl-CoA metabolism in HF patients (18). Moreover, our analyses demonstrated that the protein levels of several enzymes associated with succinyl-CoA metabolism, which may either increase or decrease succinyl-CoA levels, were changed substantially in chronic HF (Fig. 4*G*). On the other hand, our results of 5-ALA administration to MI mice support the notion that excess heme synthesis in chronic HF is one cause that leads to a reduction in succinyl-CoA. However, most succinyl-CoA-associated metabolic pathways that were found to be altered in MI mice may be closely associated with each other, and it remains unknown as to what other changes are closely associated with the decreased succinyl-CoA levels.

The myocardium of MI mice was often found to have increased heme levels, together with increased Alas1 levels. In this regard, although the increase in heme levels in the failing hearts of mice has been reported previously, the up-regulation of Alas1 is a finding (19). Moreover, the ADP/ATP-associated succinyl-CoA synthase levels were often selectively decreased in MI mice. In agreement with our mouse experiments, increased levels of *ALAS1* mRNA and decreased levels of *SUCLG1/SUCLA2* mRNA are observed in the myocardium of some HF patients (20, 21). Therefore, the molecular mechanism as to how these mRNA levels are altered in MI mice, as well as in HF patients, awaits clarification. Whether mechanisms other than those regulating mRNA levels are involved in the regulation of the protein levels of these enzymes also requires clarification in the future.

The myocardium of MI mice was also often found to have increased ketolysis, together with increased Oxct1 levels. Enhanced ketolysis indeed occurs in the failing hearts of patients, as mentioned above, and moreover, ketolysis in the heart occurs even in healthy conditions, such as during strenuous exercise (22), although it is not known whether Oxct1 levels are also altered. The up-regulation of ketogenesis and β-OHB synthesis in the liver during strenuous exercise appears to be a strategy to supply β-OHB to the brain, as well as to the heart, skeletal muscles, and kidneys as an energy source, at times when blood glucose is greatly consumed. However, although enhanced ketolysis in the heart is a normal biological phenomenon, this can be disadvantageous for failing hearts, because enhanced ketolysis requires a large amount of succinyl-CoA, despite succinyl-CoA consumption being often increased for the synthesis of heme in failing hearts. On the other hand, cardiac muscle can use fatty acids as an energy source, and it has been reported that blood levels of fatty acids are not particularly reduced in patients with HF or in mouse models (13, 23). The brain cannot take in fatty acids due to the blood–brain barrier. Furthermore, ketolysis in the heart may be a zero-sum game with respect to succinyl-CoA, because in ketolysis, succinyl-CoA is first consumed to synthesize acetoacetyl-CoA, which is then converted to acetyl-CoA and may be used to synthesize succinyl-CoA. Thus, the increased ketolysis in MI mice may or may not be causative to the decreased succinyl-CoA levels. Why failing hearts predominantly use ketone bodies rather than fatty acids, and whether this is a simple remnant of a normal system or may have some potential advantages as a biological defense system, are issues that require clarification in the future.

In this study, we treated MI mice with 5-ALA alone, whereas in most previous studies, such as in neuroscience and in oncology, 5-ALA was administered in combination with iron-containing compounds, such as sodium ferrous citrate, to promote heme synthesis (24, 25). An abnormal increase in myocardial heme is thought to further exacerbate failing hearts, such as it may increase free heme (26). On the other hand, we have shown that 5-ALA alone can compensate considerably for the consumption of succinyl-CoA without further increasing heme in the myocardium of MI mice.

In summary, we demonstrated that nutritional intervention that compensates for the altered succinyl-CoA metabolism in chronic HF (i.e., 5-ALA administration) is a promising method to treat this disease, although our results also suggested that 5-ALA by itself might not be sufficient to effectively treat HF. Therefore, our results, as well as a further understanding of the detailed metabolic changes that occur in chronic HF and the molecular mechanisms therein involved, will contribute to the development of HF therapeutics, particularly to the development of more natural treatments, as well as to the prevention of HF (27). Succinyl-CoA is the most abundant acyl-CoA in the heart (28). Whether the histone acylations involved in epigenetic control (9) are altered in chronic HF should also be clarified in the future (29).

## Materials and Methods

### Animal Procedures and Ethics Statement.

Animal experiments were performed according to a protocol approved by the Animal Care Committee of Hokkaido University (study approval 16–0115). C57BL/6J mice were used in all experiments, and were housed under standard conditions (temperature: 23–25 °C, and humidity: 40–60%) on a 12-h light/dark cycle.

### In Vivo HF Mouse Model.

As an in vivo HF model, a coronary ligated model was used, as described previously (7). Permanent left anterior descending (LAD) coronary artery ligation was performed on the mice. Male C57BL/6J mice (9–12 wk old; CLEA Japan) were anesthetized with an intraperitoneal injection of a mixture of 0.3 mg/kg body weight of medetomidine (Kyoritsu, Dorbene), 4.0 mg/kg body weight of midazolam (Astellas, Dormicum), and 5.0 mg/kg body weight of butorphanol (Meiji, Vetorphale) (MMB mixture), and then intubated and ventilated with air (supplemented with oxygen) using a small-animal respirator. The adequacy of anesthesia was monitored by the pedal withdrawal reflex. A thoracotomy was performed in the fourth left intercostal space. Then, the left ventricle was visualized and the pericardial sac was ruptured to expose the LAD coronary artery. The LAD was permanently ligated using a 4–0 Prolene suture (Covidien, Sofsilk VS-709). The suture was passed ∼0.5 mm below the tip of the left auricle. Sham-operated mice, which were used as controls, underwent thoracotomy of the heart as in coronary-ligated mice, except that their arteries were not tied. The thoracotomy was closed with 8–0 Prolene sutures (Akiyama Medical MFG, M6-80B2). The endotracheal tube was removed once spontaneous respiration resumed, and the mice were placed in a warm recovery cage maintained at 37 °C until they were completely awake. In our HF mouse model, most deaths within 24 h after MI appeared to be surgical-related, and were excluded in this study (30). After 4 wk, mice were subjected to analyses. For biochemical analysis, mice were killed with an intraperitoneal injection of the MMB mixture, and the heart was then excised and subjected to analyses. Regarding the LVs of HF mice, the infarct areas were excluded from the biochemical analyses, and only noninfarct areas were used for the experiments. Blood samples of the mice were collected from the inferior vena cava before euthanization by deep anesthesia with the MMB mixture, as described previously (31).

### Metabolite Extraction from Mouse Heart.

Mice were killed by cervical dislocation under general anesthesia. Mouse LVs, excluding the infarcted areas and the border zones between the infarcted and noninfarcted areas, were isolated within 2 min after cervical dislocation, and rapidly cryopreserved with liquid nitrogen. Pieces of the mouse LV (40–50 mg) were snap-frozen in liquid nitrogen and crushed using a MultiBeads Shocker (Bio Medical Science) at 2,000 rpm for 10 s The crushed powder was then dissolved in 1 mL of ice-cold 80% methanol per 100 mg tissue weight, sonicated five times (five rounds of sonication for 30 s and cooling for 30 s) using a BIORUPTOR (Cosmo Bio), and centrifuged at 21,500 × *g* for 5 min at 4 °C. The supernatants were then collected and subjected to LC-MS analyses.

### Metabolite Analysis by LC-MS.

To measure the TCA cycle intermediates, heart lysate supernatants (equivalent to ∼1.5 mg of the heart) were applied and separated on an ACQUITY BEH C18 column (100 mm × 2.1 mm, 1.7 μm, Waters, 186002352) and analyzed using an LCMS 8040 instrument (Shimadzu) by multiple reaction monitoring of 98 specific negative ions, as described previously (32). The mobile phase consisted of 15 mM acetic acid and 10 mM tributylamine in 3% methanol solution (A) and 100% methanol (B). The gradient elution program was as follows: 0–6 min, 0% B; 6–26 min, 0–90% B; decreased to 0% B and maintained until 15 min. The flow rate was 0.3 mL/min, and the column oven temperature was 40 °C. Parameters for the negative electrospray ionization mode were as follows: drying gas flow rate, 15 L/min; nebulizer gas flow rate, 3 L/min; heating gas flow rate, 10 L/min; desolvation line temperature, 250 °C; and heat block temperature, 400 °C; collision induced dissociation gas, 230 kPa. Data processing was performed using LabSolutions software (Shimadzu) and signal intensities were standardized against the signals of MES.

### Isolation of Mitochondria from Mouse Heart.

Mitochondria from mouse LVs, excluding the infarcted areas and the border zones between the infarcted and noninfarcted areas, were isolated as described previously (10). Briefly, excised mouse hearts were quickly minced using scissors for 4 min, and incubated with 0.1 mg/mL proteinase (Sigma-Aldrich, P8038) for 2 min at 4 °C in mitochondrial isolation buffer containing 50 mM Tris × HCl (pH 7.4), 100 mM KCl, 100 mM sucrose, 1 mM KH_2_PO_4_, 0.1 mM EGTA, and 0.2% bovine serum albumin. They were then gently homogenized using a motor-driven Teflon pestle in a glass chamber (Wheaton, 358039) with six strokes, and centrifuged at 700 × *g* for 10 min. Supernatants were recentrifuged at 10,000 × *g* for 10 min, and pellets were then suspended in the mitochondrial isolation buffer, after washing briefly once with the same buffer, and centrifuged again at 7,000 × *g* for 3 min. The resulting pellets were resuspended in a buffer containing 10 mM Tris × HCl (pH 7.4), 225 mM mannitol, 75 mM sucrose, and 0.1 mM EDTA, and subjected to analyses. All procedures, except for the preincubation with proteinase, were performed at 4 °C. Protein concentrations of the samples were measured using the bicinchoninic acid assay (Thermo Fisher Scientific, 23225).

### Immunoblotting Analysis.

Immunoblotting analysis was performed as described previously (33). Briefly, mitochondrial proteins were denatured in Laemmli buffer (100 mM Tris × HCl [pH 6.8], 4% SDS, 4% glycerol, 0.05% bromophenol blue, 12% 2-mercaptoethanol) at 100 °C for 5 min, separated by SDS-PAGE using Any kDa Criterion precast gels (Bio-Rad, 5671122J10), and transferred to polyvinylidene fluoride membranes (Bio-Rad, cat. no. 1704156). Membranes were then blocked for 1 h at room temperature in TBS-T buffer (0.1%Tween-20 in 1× PBS) containing 3% milk, and incubated with primary antibodies diluted in TBS-T with 3% milk, overnight at 4 °C. Primary antibodies used were as follows: Sucla2 (Abcam, ab202582, 1:1,000 dilution), Suclg1 (Abcam, ab204432, 1:1,000 dilution), Suclg2 (Abcam, ab241375, 1:1,000 dilution), Glud1 (Abcam, ab 168352, 1:1,000 dilution), Ogdh (Abcam, ab137773, 1:1,000 dilution), Alas1 (Abcam, ab84962, 1:1,000 dilution), Oxct-1 (Abcam, ab105320, 1:1,000 dilution), Pcca (Abcam, ab187686, 1:1,000 dilution), Mcm (Abcam, ab134956, 1:1,000 dilution), and succinyllysine (PTM BIO, PTM419, 1:1,000 dilution). After three washes with TBS-T, membranes were then incubated with a horseradish peroxide-conjugated secondary antibody (Abcam, ab97051, 1:20,000 dilution, or Santa Cruz, sc-2314, 1:5,000 dilution) in TBS-T with 3% milk, for 1 h at room temperature. After washing, peroxide-conjugated Abs retained on membranes were visualized with the enhanced chemiluminescence kit (Thermo Fisher Scientific, 32106 or 34075) coupled with ChemiDoc XRS^+^ (Bio-Rad). Densities of the signals were quantified with Image J software (NIH). Expression levels of the proteins are shown as values normalized by the protein level of voltage-dependent anion channel, which is a representative mitochondrial marker (Cell Signaling Technology, 4866, 1:1,000 dilution), or total amounts of proteins measured by Coomassie brilliant blue (CBB) staining (Bio-Rad, 1610786) or Ponceau-S staining (Beacle, BCL-PSS-01).

### Measurement of Mitochondrial OXPHOS Activities.

To assess mitochondrial OXPHOS, respiration capacities of isolated mitochondria were measured with a high-resolution respirometer (Oxygraph-2k, Oroboros) (7). Isolated mitochondria (∼30–100 µg) were placed into the respirometer chamber, filled with 2 mL of MiR05 medium (110 mmol/L sucrose, 60 mmol/L K-lactobionate, 0.5 mmol/L EGTA, 0.1% BSA, 3 mmol/L MgCl_2_, 20 mmol/L taurine, 10 mmol/L KH_2_PO_4_, 20 mmol/L Hepes, pH 7.1), and then incubated with chemicals at 37 °C in the following order (final concentrations are indicated): 1) 2 mM malate, 5 mM pyruvate, 10 mM glutamate (complex I-linked substrates), 2) 10 mM ADP, and 3) 10 mM succinate (complex II-linked substrate) and 0.5 µM rotenone (a complex I inhibitor). The O_2_ consumption rates (i.e., respiration rates) were expressed as O_2_ flux normalized to the mitochondrial protein concentration (µg/µL). DatLab software (Oroboros) was used for data acquisition and analysis.

### Measurement of Mitochondrial Heme.

Intramitochondrial heme was quantified using the QuantiChrom Heme Assay Kit (BioAssay Systems, DIHM-250), according to the manufacture’s instruction. Briefly, isolated mitochondria were mixed with 200 μL of reaction mixture provided with the assay kit, and incubated for 5 min at room temperature. Optical densities at 400 nm were then measured in a microtiter plate reader (Multiskan GO, Thermo Fisher), in which the standard curve was prepared using a heme standard provided by the assay kit.

### Measurement of Plasma β-OHB.

β-OHB levels in the plasma were measured using the β-OHB Assay Kit (Abcam, ab180876), according to the manufacture’s instruction.

### Feeding Mice with 5-ALA.

Sham or MI mice were divided into two groups after the operation; one group was given normal-water, and the other was given water containing 50 mg/L 5-ALA (Sigma Aldrich, A3785), and the amount of 5-ALA ingested was 6–8 mg/kg body weight per day. After 4 wk, mice were subjected to echocardiography and treadmill running and then killed with an intraperitoneal injection of the MMB mixture, for subsequent biochemical analyses.

### Echocardiography.

Echocardiographic measurements were performed in mice in the conscious state to avoid any effects of anesthesia on cardiac function, as described previously (33). Briefly, a commercially available echocardiography system (Toshiba Medical Systems, Aplio300) was used with a dynamically focused 12-MHz linear array transducer and a depth setting of 2.0 cm. The fur on the chest was removed using depilatory cream and a layer of acoustic coupling gel was applied to the thorax. A two-dimensional parasternal short-axis view was obtained at the levels of the papillary muscles. In general, the clearest views were obtained with the transducer lightly applied to the mid-upper left anterior chest wall. The transducer was then gently moved in the cephalad or caudad direction, and angulated until desirable images were obtained. After confirmation that the imaging was on the axis, two-dimensional targeted M-mode tracings were recorded at a paper speed of 50 mm/s. The following indexes were analyzed using the software in the echo instrument: LVEDD, LV end-systolic diameter (LVESD), percent fractional shortening (%FS), heart rate (HR), anterior wall thickness (AWT), and posterior wall thickness (PWT) of the LV.

### Treadmill Running.

Mice were subjected to treadmill running to assess their whole-body exercise capacity, as previously described (31). Briefly, each mouse was made to run on a motor-driven treadmill enclosed within a chamber with constant airflow, in which the O_2_ and CO_2_ fractions were monitored (Oxymax 2; Columbus Instruments). After a 10-min warm-up at 6 m/min at 0° inclination, the treadmill angle was fixed at 10° and the speed was gradually increased by 2 m/min until the mouse attained exhaustion. Exhaustion was defined as spending more than 10 s on the electrical shocker plate without attempting to go back onto the treadmill. Work as whole-body exercise capacity was defined as the product of the vertical running distance to exhaustion, and body weight.

### Protein Succinylation of Mitochondria.

Mitochondria isolated from mouse cardiac muscles were denatured in buffer (1% SDS and 0.07% 2-mercaptoethanol in PBS) and boiled for 10 min. Mitochondrial proteins were then incubated with an anti-succinyllysine antibody conjugated with agarose beads (PTM Biolabs, PTM419) at 4 °C with gentle rotation overnight. After washing three times with buffer (0.05% Lauryl Maltoside in PBS), the beads were dissolved in Laemmli buffer and boiled for 5 min. Supernatants were then collected after brief centrifugation, and subjected to MS/MS. After reduction with 10 mM TCEP (FUJIFILM Wako Chemicals, 203–20153) at 100 °C for 10 min and alkylation with 50 mM iodoacetamide (FUJIFILM Wako Chemicals, 093–02152) at ambient temperature for 45 min, protein samples were subjected to SDS-PAGE. Electrophoresis was stopped at the migration distance of 2 mm from the top edge of the separation gel. After CBB-staining, protein bands were excised, destained, and cut finely prior to in-gel digestion with Trypsin/Lys-C Mix (Promega, V5072) at 37 °C for 12 h. The resulting peptides were extracted from gel fragments and analyzed with Orbitrap Fusion Lumos mass spectrometer (Thermo Scientific) combined with UltiMate 3000 RSLC nano-flow HPLC (Thermo Scientific). Peptides were enriched using μ-Precolumn (0.3 mm inner diameter [i.d.] × 5 mm, 5 μm, Thermo Scientific, 160454) and separated on an AURORA column (0.075 mm i.d. × 250 mm, 1.6 μm, Ion Opticks Pty, AUR25075C18AC) using the following two-step gradient: 2–40% acetonitrile for 110 min, followed by 40–95% acetonitrile for 5 min in the presence of 0.1% formic acid. The analytical parameters of Orbitrap Fusion Lumos were set as follows: resolution of full scans = 50,000, scan range (*m/z*) = 350–1,500, maximum injection time of full scans = 50 ms, AGC target of full scans = 4 × 10^5^, dynamic exclusion duration = 30 s, cycle time of data dependent MS/MS acquisition = 2 s, activation type = HCD, detector of MS/MS = ion trap, maximum injection time of MS/MS = 35 ms, AGC target of MS/MS = 1 × 10^4^.

The MS/MS spectra were searched against the *Mus musculus* protein sequence database in SwissProt using Proteome Discoverer 2.4 software (Thermo Scientific), in which peptide identification filters were set at “false discovery rate <1%.” Label-free relative quantification analysis of proteins was performed with the default parameters of Minora Feature Detector node, Feature Mapper node, and Precursor Ions Quantifier node in Proteome Discoverer 2.4 software.

### Statistics.

Data are expressed as the mean ± SEM. Statistical analyses were performed using the unpaired Student *t* test for comparisons between two groups and by ANOVA followed by the Tukey test for comparisons between three groups, using GraphPad Prism 6 software (GraphPad). Dunnett’s method was used for multiple comparisons with a control group. For all animal experiments, all stated replicates are biological replicates. Kaplan-Meier analysis with the log-rank test was performed to compare survival rates among two groups for 28 d MI-post-surgery. For 5-ALA treatment studies, mice were randomly assigned to treatment groups. For mass spectrometry analyses, samples were processed in random order and experimenters were blinded to the experimental conditions. All experiments were successfully repeated with similar results at least two or three times. Significances between groups were indicated by asterisks (*P < 0.05).

## Supplementary Material

Supplementary File

## Data Availability

All study data are included in the article and/or *SI Appendix*.
