# Supplementary material for: Succinyl-CoA-based energy metabolism dysfunction in chronic heart failure
Source: Proc Natl Acad Sci U S A. 2022 Oct 6;119(41):e2203628119. doi: 10.1073/pnas.2203628119 (PMC9564216; doi:10.1073/pnas.2203628119)
Supplement: Supplementary File [file pnas.2203628119.sapp.pdf]

Supporting Information for

Succinyl-CoA-based energy metabolism dysfunction in chronic heart failure.

Shingo Takada[a,b,c,1, 2], Satoshi Maekawa[a,1], Takaaki Furihata[a], Naoya Kakutani[a], Daiki Setoyama[d], Koji Ueda[e], Hideo Nambu[a], Hikaru Hagiwara[a], Haruka Handa[b], Yoshizuki Fumoto[b], Soichiro Hata[b], Tomoka Masunaga[g], Arata Fukushima[a], Takashi Yokota[a], Dongchon Kang[d,f], Shintaro Kinugawa[a,g,h,2], and Hisataka Sabe[b, i,2]

[a] Department of Cardiovascular Medicine, Hokkaido University Graduate School of Medicine, Sapporo, Japan

[b] Department of Molecular Biology, Hokkaido University Graduate School of Medicine, Sapporo, Japan

[c] Department of Lifelong Sport, School of Sports Education, Hokusho University, Ebetsu, Japan

[d] Department of Clinical Chemistry and Laboratory Medicine, Kyushu University, Fukuoka, Japan

[e] Cancer Precision Medicine Center, Japanese Foundation for Cancer Research, Tokyo, Japan

[f] Clinical Laboratories, Kyushu University Hospital, Fukuoka, Japan

[g] Department of Cardiovascular Medicine, Faculty of Medical Sciences, Kyushu University, Fukuoka, Japan

[h] Division of Cardiovascular Medicine, Research Institute of Angiocardiology, Faculty of Medical Sciences, Kyushu University, Fukuoka, Japan

[i] Institute for Genetic Medicine, Hokkaido University, Sapporo, Japan.

[1] These authors contributed equally to this work

[2] To whom correspondence may be addressed.

Shingo Takada, PhD

E-mail: s-takada@hokusho-u.ac.jp; s-takada@hotmail.co.jp

Shintaro Kinugawa, MD, PhD

E-mail: kinugawa.shintaro.786@m.kyushu-u.ac.jp

Hisataka Sabe, Ph.D.

E-mail: sabeh@med.hokudai.ac.jp; sabeh@igm.hokudai.ac.jp

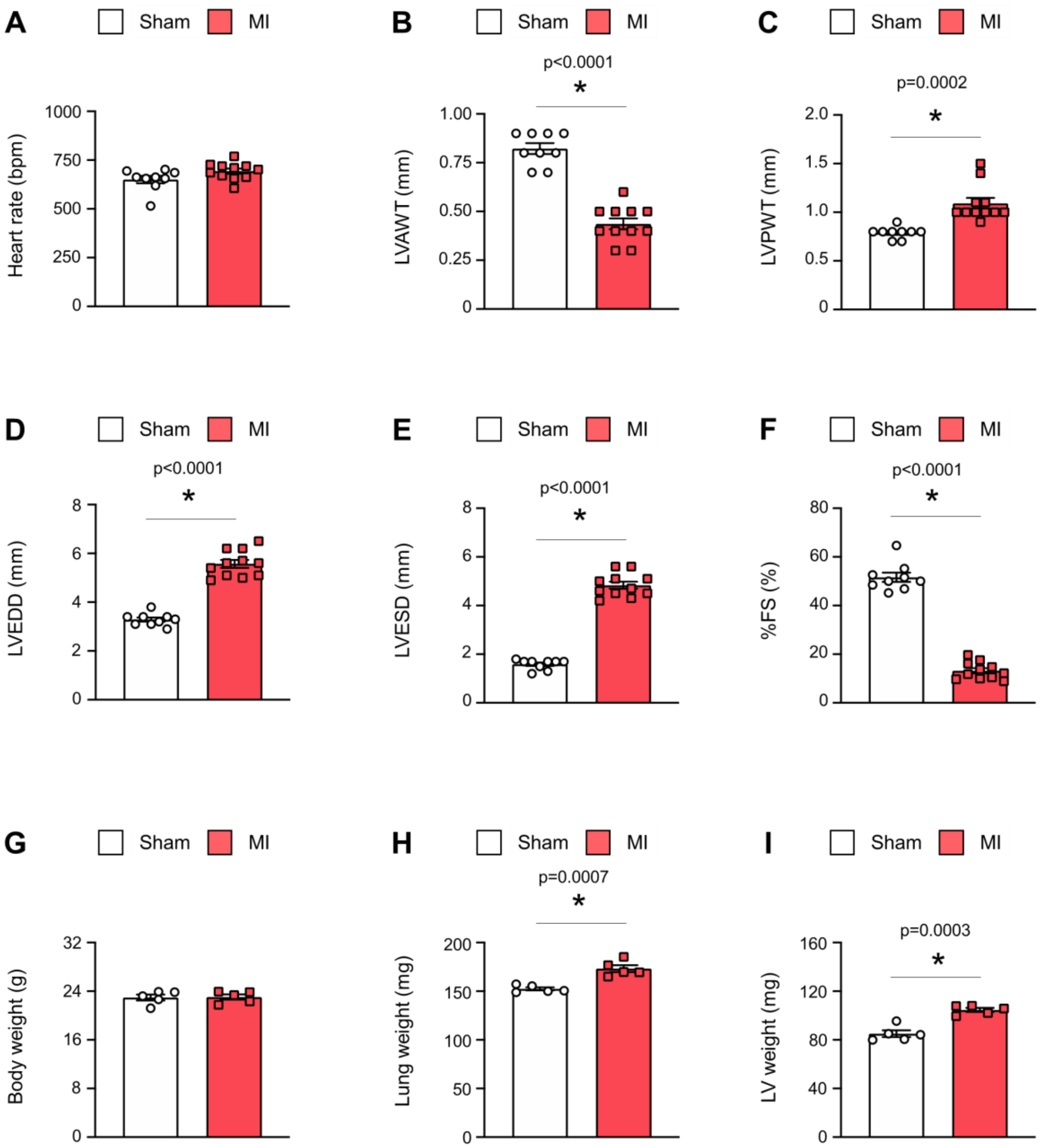

Figure S1. Cardiac parameters of the MI mice and sham mice used in this study. MI mice and sham mice used in our experiments were randomly sampled, and their cardiac parameters, namely heart rate (**A**), left ventricular (LV) anterior wall thickness (LVAWT) (**B**), LV posterior wall thickness (LVPWT) (**C**), LV end-diastolic diameter (LVEDD) (**D**), LV end-systolic diameter (ESD) (**E**), percent fractional shortening (%FS) (**F**), body weight (**G**), lung weight (**H**), and LV weight (**I**). Each data point in the dot plot

represents one individual mouse sample. Data are shown as the mean ± s.e.m. Significances between groups were tested using the unpaired *t*-test, and indicated by asterisks (*$P < 0.05$).

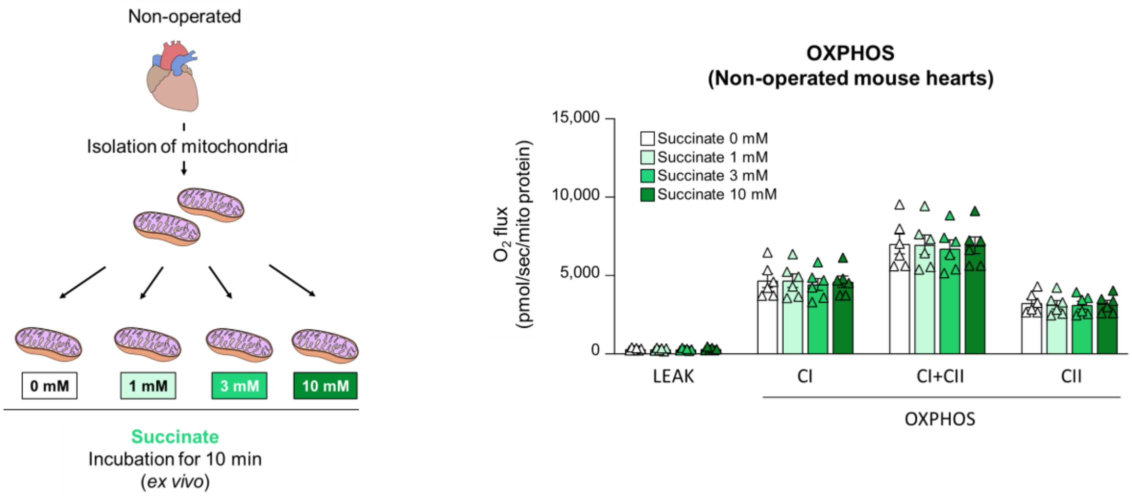

Fig. S2. Succinate does not affect OXPHOS capacities of myocardial mitochondria *ex vivo*. Experimental scheme to measure myocardial mitochondrial OXPHOS capacities in non-operated mice in response to the addition of succinate (*left*) and the actual results (*right*). Each data point in the dot plot represents one individual mouse sample. Data are shown as the mean ± s.e.m. Data were analyzed by one-way analysis of variance (ANOVA) followed by the Dunnett *post hoc* analysis. LEAK, leak state; CI, mitochondrial complex I; CII, mitochondrial complex II; OXPHOS, oxidative phosphorylation.

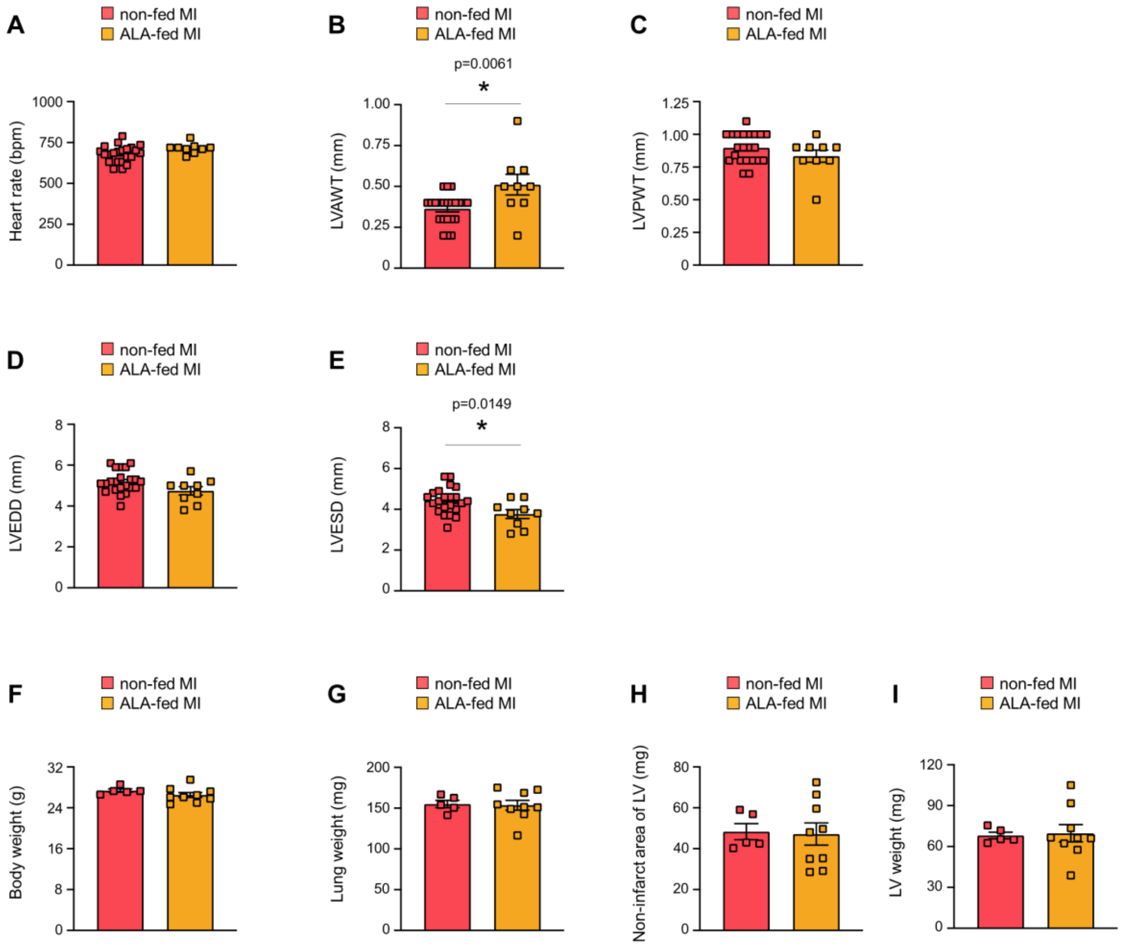

Figure S3. Cardiac parameters of 5-ALA-fed and non-fed MI mice. MI mice were administered with or without 5-ALA daily in their drinking water for 4 weeks (5-ALA-fed and non-fed, respectively), starting immediately after the coronary ligation, and then the following cardiac parameters were measured: heart rate (**A**), LVAWT (**B**), LVPWT (**C**), LVEDD (**D**), LVESD (**E**), body weight (**F**), lung weight (**G**), non-infarct area of LV weight (**H**), and LV weight (**I**). Each data point in the dot plot represents one individual mouse sample. Data are shown as the mean ± s.e.m. Significances between groups were tested using the unpaired *t*-test, and are indicated by asterisks (*$P < 0.05$).

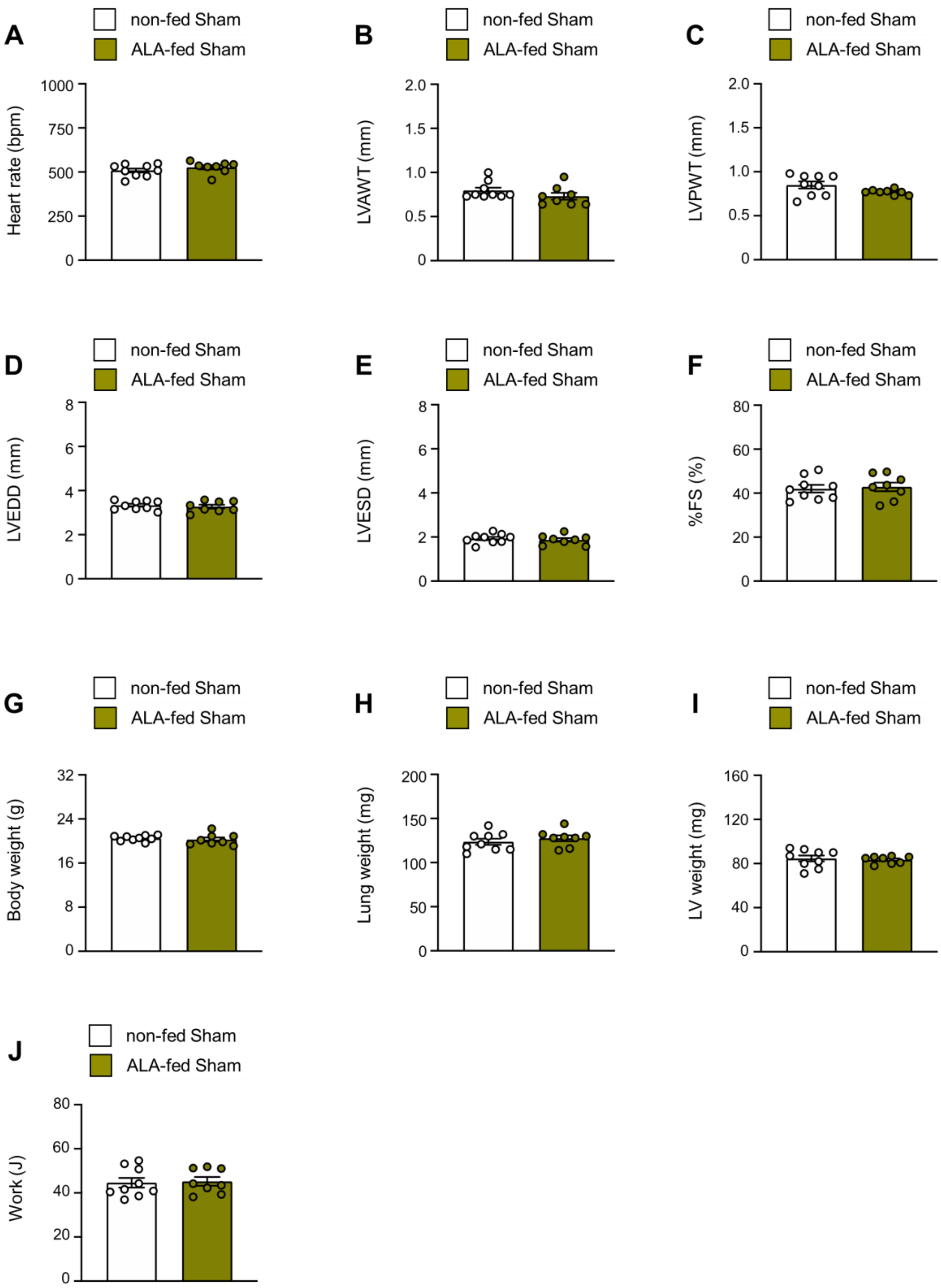

Fig. S4. Effects of 5-ALA on cardiac parameters, body weight, organ weights, and exercise capacity in sham mice. A-C, Effects of 5-ALA on cardiac parameters, body

weight, organ weights, and exercise capacity, and survival were analyzed by administering sham mice with (n = 8) or without (n = 9) 5-ALA in their drinking water for 4 weeks, starting immediately after sham operation; and then the following cardiac parameters: heart rate (**A**), LVAWT (**B**), LVPWT (**C**), LVEDD (**D**), LVESD (**E**), %FS (**F**), body weight (**G**), lung weight (**H**), LV weight (**I**), and treadmill running capacity: running distance (**J**), running time (**K**), work (**L**) were measured. Each data point in the dot plots represents one individual mouse sample. Data are shown as the mean ± s.e.m. Significances between groups were tested using the unpaired *t*-test.

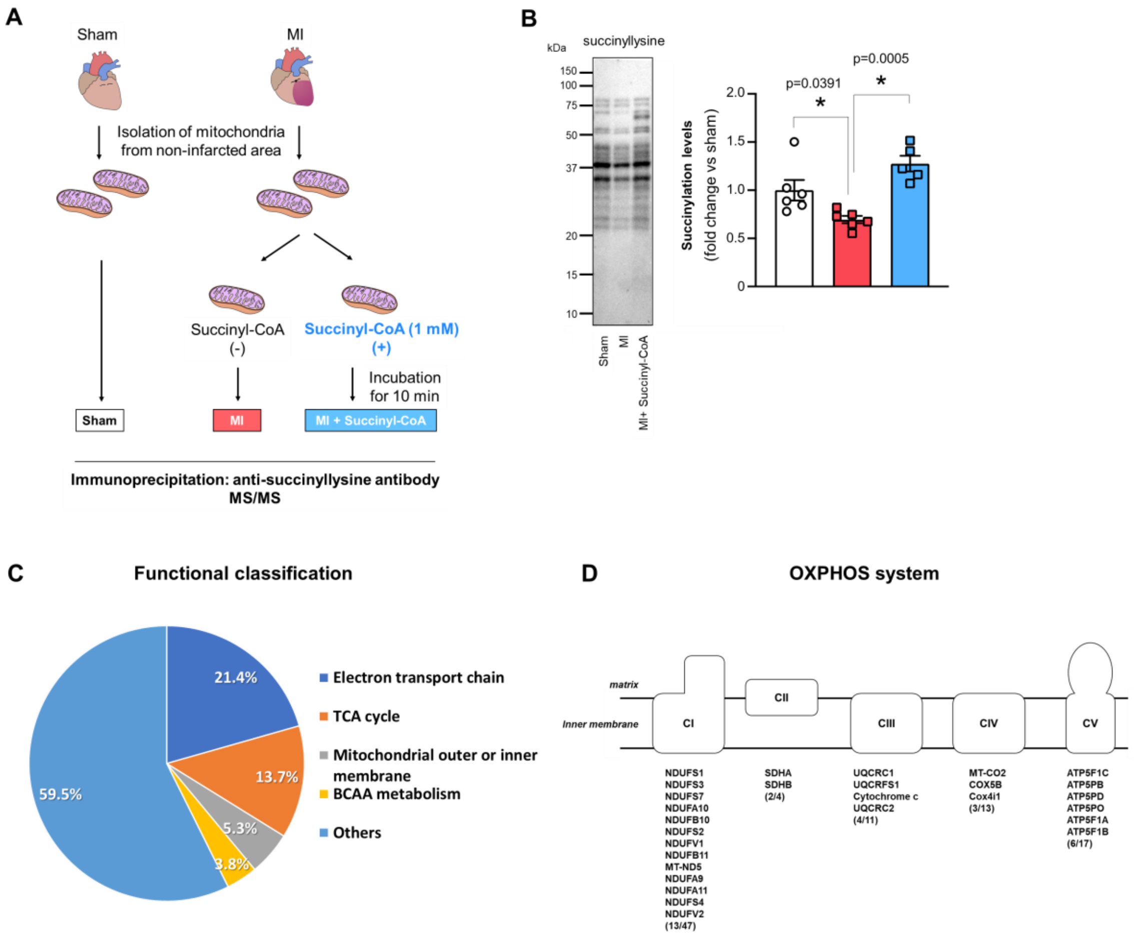
A
Sham
MI
Isolation of mitochondria
from non-infarcted area
Succinyl-CoA
(-)
Succinyl-CoA (1 mM)
(+)
Incubation
for 10 min
Sham
MI
MI + Succinyl-CoA
Immunoprecipitation: anti-succinyllysine antibody
MS/MS
B
kDa
succinyllysine
150
100
75
50
37
20
15
10
Sham
MI
MI+ Succinyl-CoA
Succinylation levels
(fold change vs sham)
p=0.0391
p=0.0005
*
*
2.0
1.5
1.0
0.5
0
C
Functional classification
21.4%
13.7%
5.3%
3.8%
59.5%
Electron transport chain
TCA cycle
Mitochondrial outer or inner
membrane
BCAA metabolism
Others
D
OXPHOS system
matrix
Inner membrane
CI
CII
CIII
CIV
CV
NDUFS1
NDUFS3
NDUFS7
NDUFA10
NDUFB10
NDUFS2
NDUFV1
NDUFB11
MT-ND5
NDUFA9
NDUFA11
NDUFS4
NDUFV2
(13/47)
SDHA
SDHB
(2/4)
UQCRC1
UQCRFS1
Cytochrome c
UQCRC2
(4/11)
MT-CO2
COX5B
Cox4i1
(3/13)
ATP5F1C
ATP5PB
ATP5PD
ATP5PO
ATP5F1A
ATP5F1B
(6/17)

E

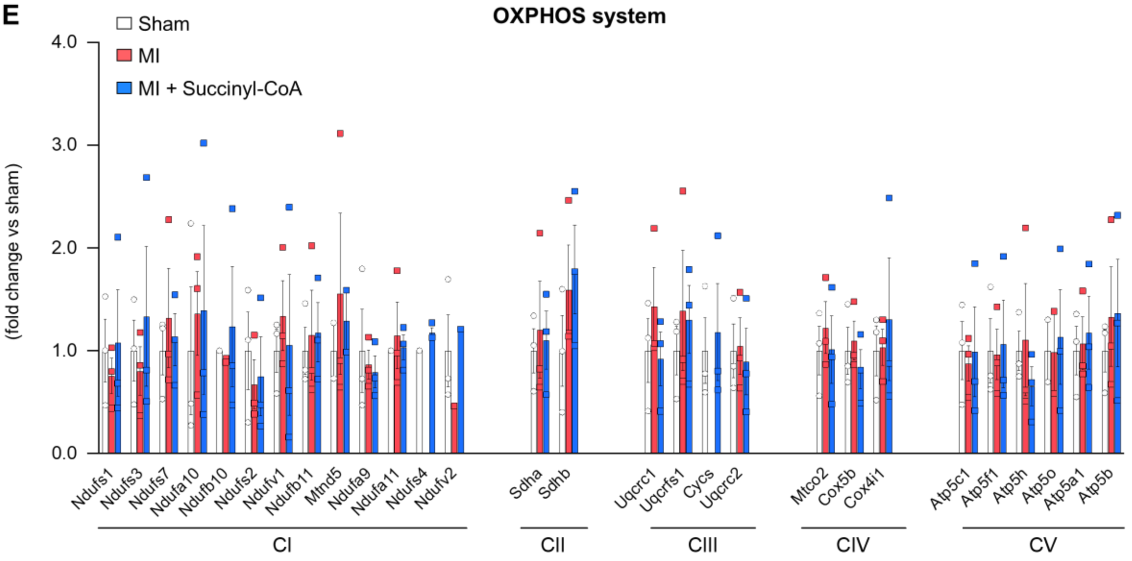
OXPHOS system
Sham
MI
MI + Succinyl-CoA
(fold change vs sham)
4.0
3.0
2.0
1.0
0.0
Ndufs1
Ndufs3
Ndufs7
Ndufa10
Ndufb10
Ndufs2
Ndufv1
Ndufb11
Mtnd5
Ndufa9
Ndufa11
Ndufs4
Ndufv2
Sdha
Sdhb
Uqcrc1
Uqcrfs1
Cycs
Uqcrc2
Mtco2
Cox5b
Cox4i1
Atp5c1
Atp5f1
Atp5h
Atp5o
Atp5a1
Atp5b
CI
CII
CIII
CIV
CV

F

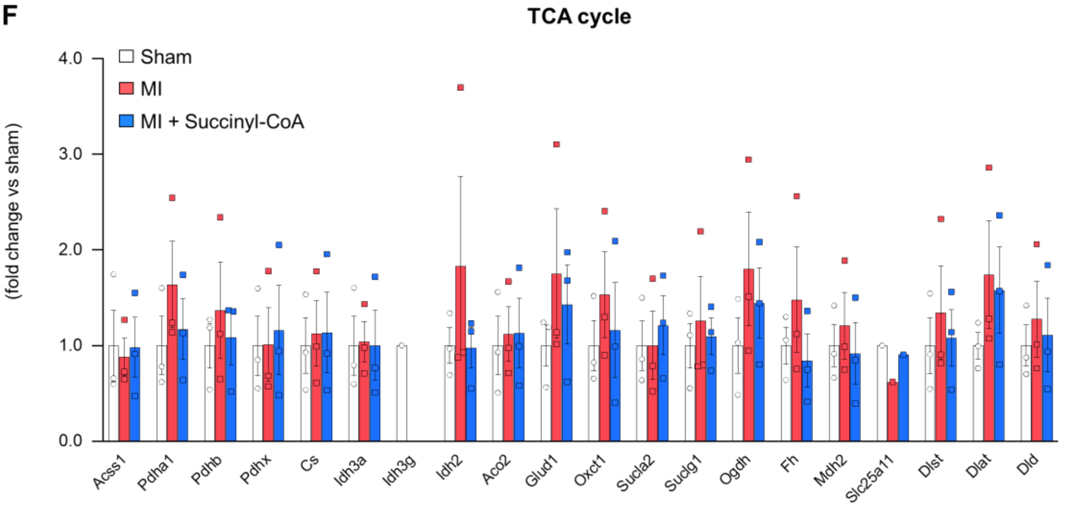
TCA cycle
Sham
MI
MI + Succinyl-CoA
(fold change vs sham)
4.0
3.0
2.0
1.0
0.0
Acss1
Pdha1
Pdhb
Pdhx
Cs
Idh3a
Idh3g
Idh2
Aco2
Glud1
Oxct1
Sucla2
Suclg1
Ogdh
Fh
Mdh2
Slc25a11
Dlst
Dlat
Dld

G

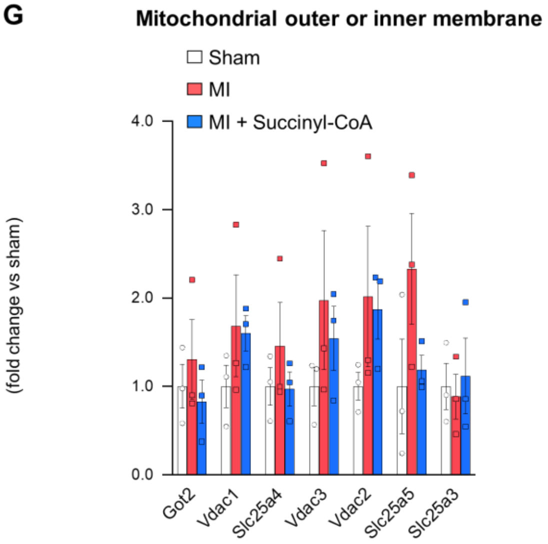
Mitochondrial outer or inner membrane
Sham
MI
MI + Succinyl-CoA
(fold change vs sham)
4.0
3.0
2.0
1.0
0.0
Got2
Vdac1
Slc25a4
Vdac3
Vdac2
Slc25a5
Slc25a3

H

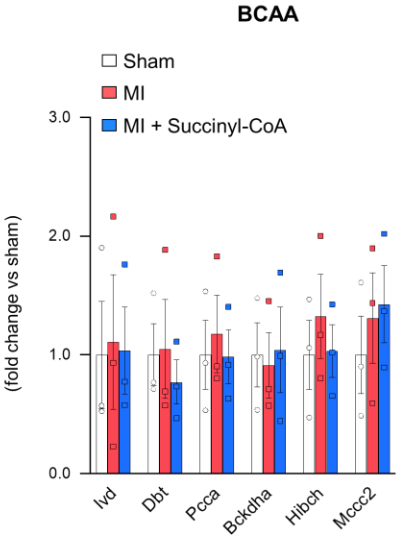
BCAA
Sham
MI
MI + Succinyl-CoA
(fold change vs sham)
3.0
2.0
1.0
0.0
Ivd
Dbt
Pcca
Bckdha
Hibch
Mccc2

Fig. S5. Perturbation of protein succinylation in myocardial mitochondria during chronic HF. A, B, Experimental scheme to compare protein succinylation in myocardial mitochondria isolated from MI mice with those from sham mice, and their changes in response to succinyl-CoA (**A**); and the actual results (**B**). In **B**, a representative anti-succinyllysine immunoblot of mitochondrial proteins after their separation by SDS-gel electrophoresis is shown on the left, and results of the quantification of all blots is shown on the right ($n = 5$ for each set of samples). **C**, Percentage of succinylated mitochondrial proteins classified based on their function. **D**, the name of each protein involved in each component of mitochondrial OXPHOS. **E-H**, Relative levels of succinylation of each myocardial mitochondrial protein in MI mice and sham mice, and changes in the succinylation of proteins of the OXPHOS system (**E**), the TCA cycle (**F**, outer and inner membrane components (**G**), and those involved in branched-chain amino acid (BCAA) metabolism (**H**) in the myocardial mitochondria of MI mice in response to the addition of succinyl-CoA. In **B, E-H**, each data point in the dot plot represents one individual mouse sample. Data are shown as the mean ± s.e.m. In **B**, data were analyzed by one-way ANOVA followed by the Tukey *post hoc* analysis. Significances between groups were indicated by asterisks ($^{*}P < 0.05$).

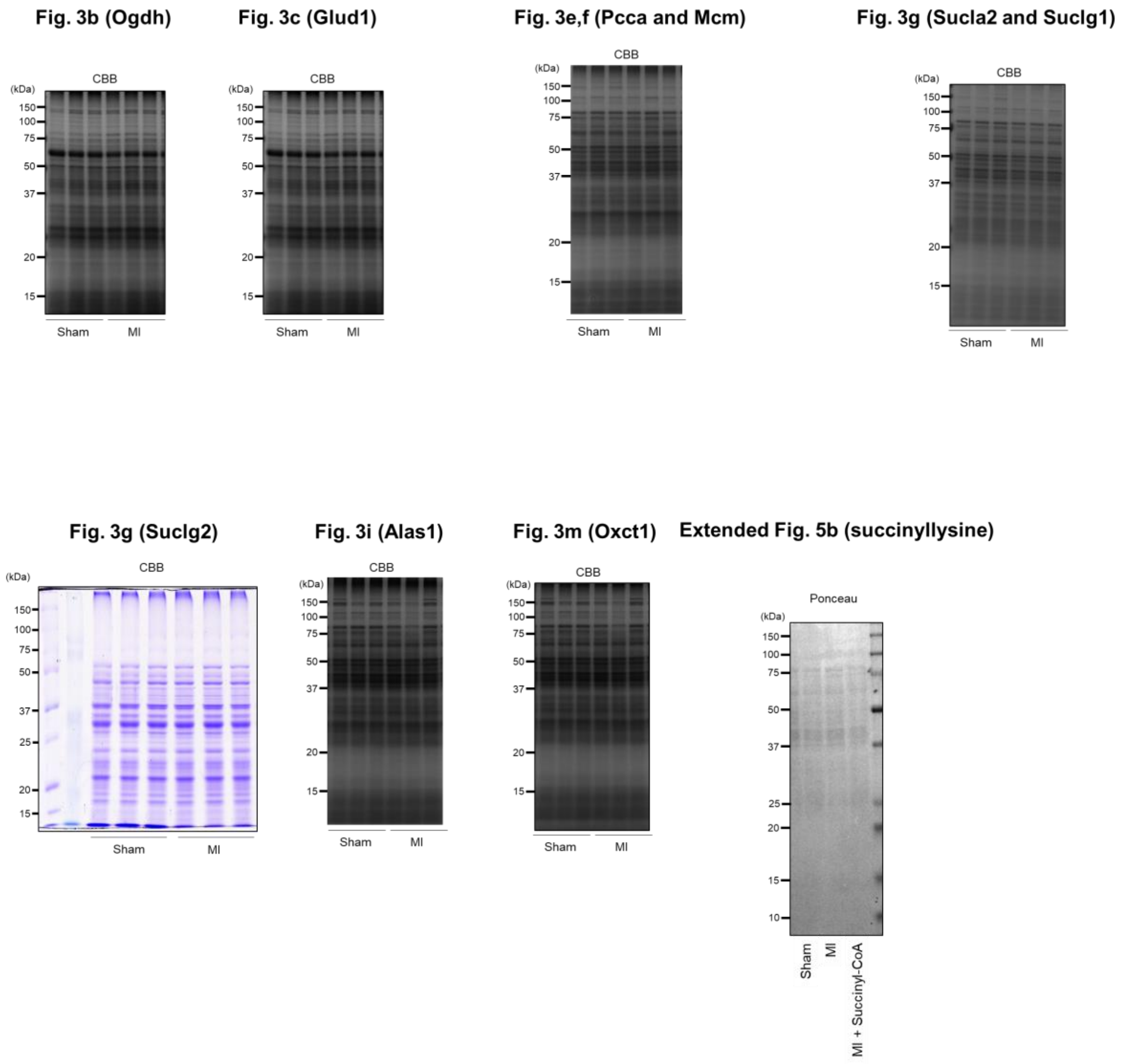

Fig. S6. Full size scans of the internal controls of the immunoblots shown in the figures of this paper.
